# Anatomical Thin Titanium Mesh Plate Structural Optimization for Zygomatic-Maxillary Complex Fracture under Fatigue Testing

**DOI:** 10.1155/2018/9398647

**Published:** 2018-05-20

**Authors:** Yu-Tzu Wang, Shao-Fu Huang, Yu-Ting Fang, Shou-Chieh Huang, Hwei-Fang Cheng, Chih-Hao Chen, Po-Fang Wang, Chun-Li Lin

**Affiliations:** ^1^Department of Biomedical Engineering, National Yang-Ming University, Taipei, Taiwan; ^2^Division of Research and Analysis, Food and Drug Administration, Taipei, Taiwan; ^3^Craniofacial Research Center, Department of Plastic and Reconstructive Surgery, Chang Gung Memorial Hospital, Linkou, Taiwan; ^4^Chang Gung University, College of Medicine, 5, Fu-Hsin Street, Kewi-shan, Taoyuan, Taiwan; ^5^Department of Plastic and Reconstruction Surgery, Chang Gung Memorial Hospital, Linkou, Taiwan

## Abstract

This study performs a structural optimization of anatomical thin titanium mesh (ATTM) plate and optimal designed ATTM plate fabricated using additive manufacturing (AM) to verify its stabilization under fatigue testing. Finite element (FE) analysis was used to simulate the structural bending resistance of a regular ATTM plate. The Taguchi method was employed to identify the significance of each design factor in controlling the deflection and determine an optimal combination of designed factors. The optimal designed ATTM plate with patient-matched facial contour was fabricated using AM and applied to a ZMC comminuted fracture to evaluate the resting maxillary micromotion/strain under fatigue testing. The Taguchi analysis found that the ATTM plate required a designed internal hole distance to be 0.9 mm, internal hole diameter to be 1 mm, plate thickness to be 0.8 mm, and plate height to be 10 mm. The designed plate thickness factor primarily dominated the bending resistance up to 78% importance. The averaged micromotion (displacement) and strain of the maxillary bone showed that ZMC fracture fixation using the miniplate was significantly higher than those using the AM optimal designed ATTM plate. This study concluded that the optimal designed ATTM plate with enough strength to resist the bending effect can be obtained by combining FE and Taguchi analyses. The optimal designed ATTM plate with patient-matched facial contour fabricated using AM provides superior stabilization for ZMC comminuted fractured bone segments.

## 1. Introduction

The mid-facial anatomy is mostly composed of bones of different thickness and forms a cavity structure. This area can be divided into maxilla, zygomatic bone, nasoorbital and nasal ethmoid sinus (nasoethmoid, NOE), and so forth. The mid-face trauma usually included and combined bones, soft tissues, and teeth damaged at the same time. For patient with nonappropriate clinical treatment, it may result in serious consequences, such as osteopetrosis, malocclusion, and visual impairment. The zygomatic-maxillary complex (ZMC) fracture, one of the severe mid-face traumas, involves fracture(s) of the zygoma or adjacent bones, such as the maxilla, orbit, or temporal bone and is the second most frequently fractured bone of the craniofacial skeleton [1]. Open reduction and rigid internal fixation surgical repair techniques involve extensive exposure and reduction of the ZMC through a combination of coronal approaches and mini-titanium plate fixation to at least 3 of the 4 buttresses is the standard goal of clinical treatment [2–4]. However, open reduction and miniplate fixation are currently the presumed state-of-the-art repair option for complex ZMC fracture reduction, easily leading to incorrect ZMC bone fracture segments position realignment, making satisfactory mid-face symmetry difficult to obtain [5, 6].

**Figure 1:**
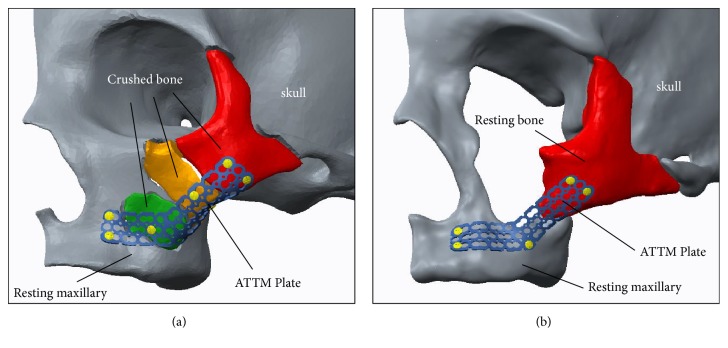
(a) The illustrations of the “L”-shape ATTM plate were anticipated to be fixed on the ZMC anterior maxilla and lateral buttress according to the patient-matched facial contour. (b) The illustrations of the optimal designed ATTM plate were projected to the desired position ZMC comminuted fracture case.

The anatomical thin titanium mesh (ATTM) regular flat plate developed based on the Asian normal ZMC image database can offer reduction guidance and fixation function with good interfacial fitness to recover the original facial contour by combining with patient-matched prebent technique (mechanical stamping) was proposed [7]. The ATTM plate was designed as an “L”-shape and screw can anticipate to be fixed on the ZMC anterior maxilla and lateral buttress according to the patient-matched facial contour (Figure 1(a)). However, the ATTM plate with patient-matched facial curvature through image projection process can be fabricated instead of using the additive manufacturing (AM) process to build complex metal parts direct from 3D CAD data. This technique has been used recently for the construction of customized artificial implants [8–11]. Despite the reduction guidance and fixation function reflected in the ATTM plate, the structural mechanical strength of the plate needs to be emphasized when used for repairing the mid-face considering the buttress characteristic. The buttresses were anatomically described as areas in the mid-facial bones which have a vertical and horizontal pillar form composed of a thick cortical bone to transfer most of the occlusal load and skull stabilization [12].

Although the finite element (FE) method provides mechanical responses and alters parameters in a more controllable manner, it becomes commonly used as an analytical tool in dental biomechanical studies [13–15]. However, when applying the FE method to investigate every possible combination of values for each parameter, the total number of simulations required is extremely high. The ATTM plate structural mechanical strength is related to several design factors, i.e., plate height, plate thickness, internal hole diameter, and hole distance. Combining FE with the statistical Taguchi method to reduce the total number of required simulations was proposed in many studies [16–18]; the Taguchi method utilizes an orthogonal array to significantly reduce the total number of required simulations and contains a well-chosen subset of all possible test condition combinations. The Taguchi method achieves a balanced comparison of the levels of any factor. This method can be combined with finite element (FE) analysis to explore the sensitivity of a model to different input parameters and thereby identify the optimal combination of designed factors [16, 19].

This study investigates the structural strength of the regular ATTM plate for multifactorial designed factors (internal hole distance, internal hole diameter, plate thickness, and plate height) with different levels using a FE approach. The Taguchi method was used to identify the importance of each design factor and suggests an optimal combination of design factors for the ATTM plate design. The optimal ATTM plate design with patient-matched facial curvature through image projection process was fabricated using an AM process and applied to a ZMC comminuted fracture to verify the method feasibility/stabilization compared to that of the same fracture case using a commercial mini-titanium plate to buttress under fatigue testing.

## 2. Materials and Methods

### 2.1. Factorial Design of the ATTM Plate

**Figure 2:**
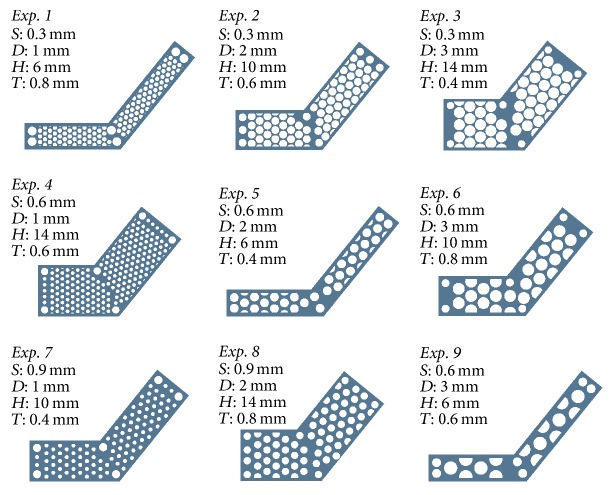
Nine solid models according to Table 1 with different designed factor combinations were derived from a CAD system.

Four design factors were considered according to the surgical approaches to evaluate the ATTM plate mechanical bending strength. These factors included internal hole distance (0.3 mm, 0.6 mm, and 0.9 mm), internal hole diameter (1 mm, 2 mm, and 3 mm), plate thickness (0.4 mm, 0.6 mm, and 0.8 mm), and plate height (6 mm, 10 mm, and 14 mm). Each factor was assigned three levels (Table 1). Originally, a total of 81 analyses (34) were required to identify the relative significance of the design factors using a full factorial approach. However, only nine simulations were required when the Taguchi method was employed. The internal hole distance, internal hole diameter, plate thickness, and plate height configurations in the ATTM plate are shown matched to an L9 orthogonal array in Figure 2 [20]. The L9 orthogonal array is comprised of 9 individual experiments as indicated by the 9 rows. The array columns represent the four factors noted in Table 1. Entries in the array represent the levels of these factors.

### 2.2. FE Analysis of the ATTM Plate and the Taguchi Method

Nine solid models (according to Figure 2 and Table 1 with different designed factor combinations) were derived from a CAD system (Creo Parametric v2.0, PTC, Needham, MA, USA). The corresponding FE mesh models were generated with quadratic ten-node tetrahedral structural solid elements after the mesh convergence test while controlling the strain energy and displacement variations < 5% for models with different element sizes. To understand the ATTM plate bending strength, a simulation test was simplified into a cantilever beam problem, with all degrees of freedom fixed on one end and 10 N downward pressure applied as the load condition on the other end for each model to perform FE analysis. The material properties were assumed to be linear, homogeneous, and isotropic and the elastic modulus/Poisson's ratio of Ti6Al4V alloy were 110 GPa/0.35 [21]. The deflections (downward displacement) of each plate were recorded to evaluate the bending resistance and the main effect at each level of all investigated factors on the displacement was computed [17]. The sum of squares for each design factor was calculated by performing an analysis of variance (ANOVA; Minitab version 12.23, Minitab Inc., PA, USA). For example, the sum of squares for a specific response to a given plate height would be equal to 3[*R*(PH_1_)  −  *R*_*m*_]^2^ + 3[*R*(PH_2_)  −  *R*_*m*_]^2^ + 3[*R*(PH_3_)  −  *R*_*m*_]^2^, where *R*(PH_1_), *R*(PH_2_), and *R*(PH_3_) are the mean plate height responses at levels 1 through 3, respectively, and *R*_*m*_ is the overall mean response over the 9 trials. To determine the relative importance of these factors, ANOVA was performed for the three-level analysis [16].

### 2.3. AM and Fatigue Testing of the Application

According to the Taguchi analysis, the optimal designed ATTM plate factor combination was set to internal hole distance as 0.9 mm, internal hole diameter as 1 mm, plate thickness as 0.8 mm, and plate height as 10 mm. The plate profile was represented similar to Exp. 7 in Figure 2 but plate thickness was set to 0.8 mm and the solid model was generated from CAD system.

**Figure 3:**
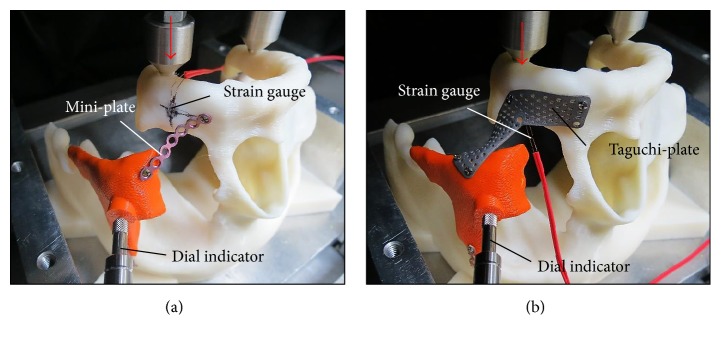
The traditional miniplate contoured manually by our surgeon and AM ATTM plates were fixed on the ZMC comminuted fracture case with ABS bone material. Samples included ABS bone models and fixation plates clamped onto a test machine with the axial load cell to perform the fatigue testing.

Fatigue testing was performed to evaluate the bone segment stabilization of the ZMC fracture fixation using the optimal designed ATTM plate and traditional solid titanium miniplate. One ZMC comminuted fracture from one female (27 years old) was selected as the test sample. The corresponding image model (with comminuted segments) was reconstructed and duplicated as the ABS (ABS-P430, Stratasys, Ltd., Minnesota, USA) plastic material using a 3D printer (Dimension 1200es SST, Stratasys, Ltd., Minnesota, USA). Three ATTM plates were projected to the desired positions (Figure 1(b)) produced by AM using a selective laser melting system (Mlab cusing R, Concept Laser Inc., Lichtenfels, Germany) of surgical Ti6Al4V alloy powder and traditional miniplate contoured manually by our surgeon. The sample plates were fixed onto the resting bone surfaces of the ZMC fracture ABS bone models. All samples included ABS bone models and fixation plates clamped onto a test machine (E3000, Instron, Canton, Mass) with the axial load cell. The fatigue tests were carried out by applying cyclic loads on the molars set at 200 N to 20 N with sine wave pattern (Figure 3). The test frequency was set at 6 Hz according to the study by Nie et al. [22]. The number of cycles at each load was recorded until 30000 cycles, which represented 1.5 months of simulated chewing function after surgery. Averaged micromotion (displacement) and strain of the maxillary bone at each of 5000 load cycles were recorded using a dial indicator and strain gage, respectively [19].

## 3. Results

**Figure 4:**
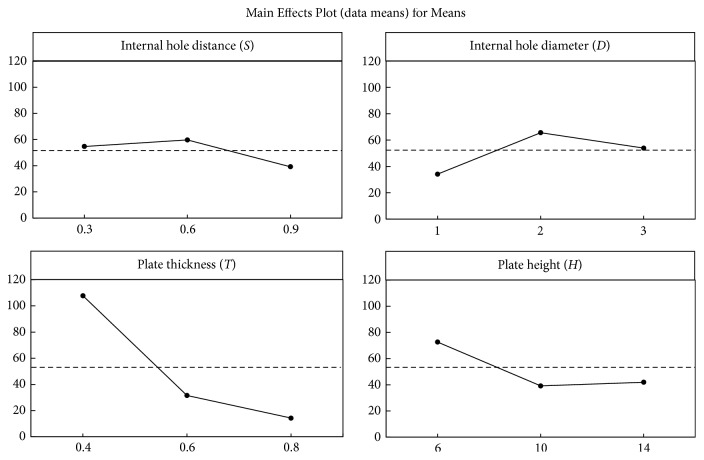
Main effects of the internal hole distance, internal hole diameter, plate thickness, and plate height at each level for ATTM plate deflection.

**Figure 5:**
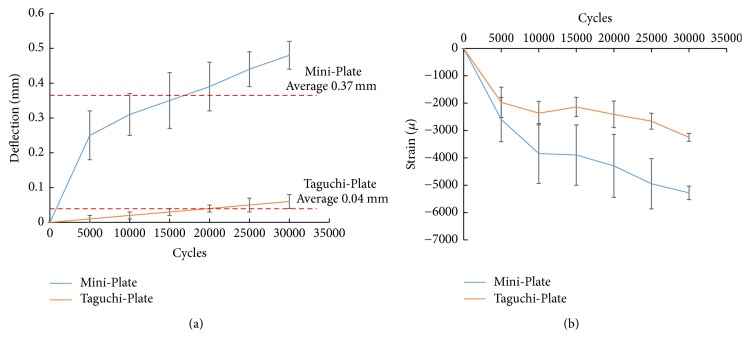
The averaged (a) micromotion (displacement) and (b) strain of the maxillary bone at each of 5000 load cycles.

Results regarding the main internal hole distance, internal hole diameter, plate thickness, and plate height effects at each level for maximal ATTM plate deflection are presented in Figure 4. The optimal designed ATTM plate with the lowest deflection and output by AM for ZMC fracture application was found as the combination of internal hole distance to 0.9 mm, internal hole diameter to 1 mm, plate thickness to 0.8 mm, and plate height to 10 mm. The relative importance of the designed factors analysis of variance (ANOVA) was indicated in that magnitude of the deflection in the ATTM plate was determined primarily by plate thickness (78%), followed by plate height (11%), internal hole diameter (8%), and hole distance (3%). The averaged micromotion (displacement) and strain of the maxillary bone at each of 5000 load cycles were recorded and presented in Figure 5 and showed that corresponding displacement and strain values of ZMC fracture fixation using a miniplate were significantly higher than those using the AM optimal designed ATTM plate at each of 5000 load cycles. The averaged maxillary bone fixation displacement using the miniplate was 9.25 times (0.37 mm/0.04 mm) that using the ATTM plate.

## 4. Discussion

A regular ATTM plate with L-shape for ZMC fracture based on the corresponding Asian medical image database was proposed in our previous work and can effectively reduce the number of traditional miniplates used. The patient-matched stamped prebent ATTM plate indeed fits the fractured bone profile and improves the overall reduction efficiency [7]. A regular ATTM plate can also be projected to the desired fixation positions on the anterior maxillary for requirements to generate a solid model in the CAD system and output as the product using AM. This technique has been widely used in several medical applications for product customization/personalization, cost-effective design, and manufacturing democratization [8–11].

However, the ATTM plate must maintain adequate bending strength to ensure that it does not deform during implantation surgery or lose plate deformation accuracy. The plate design should consider the relationship between resistance to bending and several design factors. Mid-face reconstructive surgery requires adequate load transfer to restore the functional role such as proper load transfer, as well as the aesthetic role like proper facial proportions [23]. The ATTM plate fixed on the anterior maxillary was anticipated to function similar to the zygomatic-maxillary buttress and in an uninjured skull. Therefore, the optimal structural mechanical strength in the regular ATTM plate with multifactorial designed factors was investigated in this study.

Although the FE analysis has been accepted as a good numerical tool for examining the detailed mechanical responses in many biological/engineering problems [16, 19], Dar et al. suggested that correct use of this method should facilitate more realistic modeling of both natural and synthetic systems and give a better indication of model sensitivity to the input parameters [18]. Therefore, the Taguchi method was used in combination with FE analysis to explore the sensitivity of a model to different designed factors and thereby reduce the experimental effort required to investigate multiple factors in this study. The relative importance of the investigated factors indicated that plate thickness and height were the two major factors influencing the plate deflection (bending resistance). Combined with the main effect plot, the plate deflection value decreased with increasing plate thickness and height. The ATTM plate designed with 0.8 mm thickness can provide the best bending resistance resulting from the plate cross section with the largest moment of inertia. The variation in plate height started to decrease dramatically and then converge when the plate height was greater than 10 mm. This indicates that a plate height over 10 mm offers resistance to bending. Thus, because of this bending resistance and in consideration of the limited space for surgical approach, the ATTM plate profile was set at 0.8 mm to thickness and 10 mm to height.

The ZMC comminuted fracture with resting maxillary was elected as the fatigue testing case because its structural instability is more apparent. The fatigue test results showed that the averaged micromotion (displacement) of the resting maxillary increased dramatically with the number of cycle loads and the variation between the miniplate and ATTM plate becomes bigger especially after 5000 load steps. The averaged strain value (5279 *μ*) has exceeded the critical value (>4000 *μ*) (the mechanostat theory) [24, 25] when ZMC comminuted fracture using the miniplate for fatigue testing; although current stimuli bone is ABS material, this must be paid attention.

The multidirectional load might be applied to the titanium mesh during occlusion. However, the axial load condition with 200 N applied on the molars was considered as the worst load condition, which might be the largest moment effect induced by the maximum axial force multiplied by the maximum arm. Also, the dimension of the optimal regular ATTM plate was 10 mm at height and 0.8 mm thickness with 1.3 g weight. The contour profile of the ATTM plate, that is, “L” shape for ZMC fracture, was designed based on the corresponding Asian medical image database which was applied for the MV surgical incision approach in our previous study [7]. The optimal designed ATTM plate with appropriate structural strength proposed in this study was suggested to be more suitable to use in ZMC fracture with fewer fractured segments or general comminuted fracture with enough resting maxillary to fix by fixation screws. However, serious ZMC comminuted fracture with too many small bone segments might need to consider whether there is enough bone available for screw fixation. Facial asymmetry, malocclusion, and other clinical complications might arise when the fractured segments are not reduced to the expected positions with inappropriate optimal design ATTM plate application. Further clinical testing is needed to verify if the optimal design ATTM plate conforms to the clinical requirements.

## 5. Conclusions

This study proposed an optimal designed ATTM plate with suitable structural strength to resist bending effect obtained by FE and Taguchi analyses. The optimal designed ATTM plate can be projected onto the resting maxillary geometry to recover the original facial contour and fabricated using AM. The ZMC comminuted fracture application confirms that the optimal designed ATTM plate indeed fits the fractured bone profile, improves reduction efficiency, and provides superior stabilization for fractured bone segments.

## Figures and Tables

**Table 1:** The ATTM designed factors and Taguchi L9 orthogonal array table.

Number of Exp.	Internal hole distance (*S*)	Internal hole diameter (*D*)	Plate thickness (*T*)	Plate height (*H*)
1	0.3	1	0.8	6
2	0.3	2	0.6	10
3	0.3	3	0.4	14
4	0.6	1	0.6	14
5	0.6	2	0.4	6
6	0.6	3	0.8	10
7	0.9	1	0.4	10
8	0.9	2	0.8	14
9	0.9	3	0.6	6
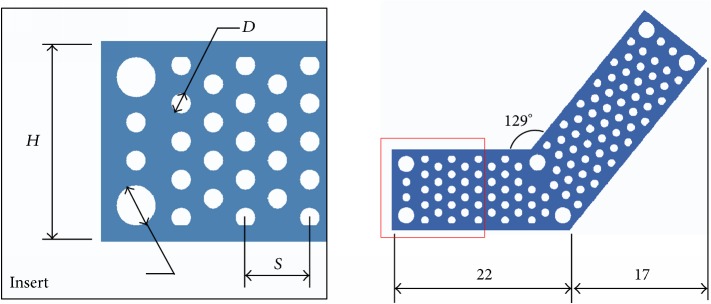				

## Data Availability

The data used to support the findings of this study are available from the corresponding author upon request.
